# Potential impact of groundnut production technology on welfare of smallholder farmers in Ghana

**DOI:** 10.1371/journal.pone.0260877

**Published:** 2022-01-14

**Authors:** Bekele Hundie Kotu, Abdul Rahman Nurudeen, Francis Muthoni, Irmgard Hoeschle-Zeledon, Fred Kizito

**Affiliations:** 1 International Institute of Tropical Agriculture, Ghana Office, Tamale, Ghana; 2 International Institute of Tropical Agriculture, Tamale, Ghana; 3 International Institute of Tropical Agriculture, Duluti, Arusha, Tanzania; 4 International Institute of Tropical Agriculture, Ibadan, Oyo State, Nigeria; International Maize and Wheat Improvement Centre: Centro Internacional de Mejoramiento de Maiz y Trigo, MEXICO

## Abstract

This study was conducted to assess the potential impact of applying a new groundnut planting density on welfare of smallholder farmers in northern Ghana. We used data from on-farm experiments, focus group discussions, and a household survey. We followed three steps in our analysis. First, we conducted cost-benefit analysis in which we showed the economic advantage of the new technology over the farmers’ practice. Second, we predicted adoption rates along timeline using the Adoption and Diffusion Outcome Prediction Tool (ADOPT). Third, using the results of the first and the second steps, we estimated the potential impact of the technology on poverty at household level using a combination of methods such as economic surplus model and econometric model. The cost-benefit analysis shows that increasing plant density increases farmers’ financial returns i.e., the benefit-cost-ratio increases from 1.05 under farmers’ practice to 1.87 under the best plant density option, which is 22 plants/sqm. The adoption prediction analysis shows that the maximum adoption rate for the best practice will be 62% which will take about nine years to reach. At the maximum adoption rate the incidence of extreme poverty will be reduced by about 3.6% if farmers have access to the international groundnut market and by about 2% if they do not have. The intervention will also reduce poverty gap and poverty severity. The results suggest that policy actions which can improve farmers’ access to the international market will enhance farmers’ welfare more than the situation in which farmers have access to domestic markets only. Furthermore, promoting a more integrated groundnut value-chain can broaden the demand base of the produce resulting in higher and sustainable impact of the technology on the welfare of groundnut producers and beyond.

## 1. Introduction

Growth in agriculture is one of the effective means to improve the welfare of poor people in developing countries as most of the poor in these countries directly depend on this sector. Studies show that investing in agriculture to increase its output can directly improve the welfare of hundreds of millions of poor in developing countries [1–4]. For instance, [4] estimates that GDP growth originating from agriculture would be at least twice as effective as the other sectors in reducing poverty. [5] estimated that the contribution of agriculture to poverty reduction in Sub-Saharan Africa would be 4.25 times the contribution of equivalent investment in the service sector. Similarly, [2] showed that a 1% increase in agricultural productivity would reduce poverty incidence by 0.72% in Africa. Other more recent studies also show that the contribution of agriculture to poverty reduction is high in developing countries [5–8]. While output growth that would lead to poverty reduction among the rural population may come through expansion of agricultural land, it is becoming more and more difficult nowadays for many countries in Sub-Saharan Africa to realize agricultural growth by this means [9, 10]. Therefore, these countries must achieve growth in agricultural output and food security through investments in agricultural research and extension to generate and disseminate productivity increasing technologies [1].

The most direct effect of a yield increasing agricultural technology is the productivity gain of the adopter whereas the indirect effects are gains derived from adoption by other farmers leading to lower food prices, employment creation, and growth linkage effects [1, 11–13]. A historical example of success in this regard is the adoption of green revolution technologies, which reduced rural poverty in developing Asia from 58.7% in 1970 to 29.9% in the 1990s through yield gains and expansion of rural employments [13]. Recent studies also show that adoption of improved agricultural technologies can reduce rural poverty substantially [8, 12, 14, 15]. However, the impact of technologies on the welfare of the adopters, non-adopter producers, and general consumers is not uniform. Adopters are expected to benefit from yield increments and per-unit cost reductions while consumers may benefit from the decrease in market prices associated with the outward shift in output supply [16]. However, non-adopter producers may lose benefit because of output price reductions. The benefits/loss accruing to each of the economic actors would vary depending on different factors such as the extent of reduction in per-unit cost of production induced by the new technology, the structure and scope of product and input markets, and the degree to which the target product has been commercialized among the adopting farmers [14, 16–18].

In this study, we contribute to the discussion about the linkage between poverty and technology adoption in agriculture by presenting a case study on groundnut production technology in Ghana. Groundnut plays a significant role in household consumption and income in Ghana [19, 20]. Moreover, as a legume crop, it plays an important role in improving soil fertility and conserving soil moisture thereby contributing to the reduction of households’ vulnerability to weather-related shocks while improving post-shock resilience [21–24]. Nevertheless, its yield from smallholder farms is less than 50% of its potential yield [25]. While several factors contribute to the substantial yield gap observed in groundnut production, the low plant density which farmers use (i.e., about 9 plants /sqm) is one of the key factors [19, 26]. Several studies in Ghana and other places have shown that applying plant densities which are higher than the farmers’ practice have substantially increased grain yield [26–29]. For instance, [26] and [29] show that yield can be doubled by doubling the current planting density of the farmers. However, most farmers in northern Ghana still plant groundnut at a density of about 9 plants/sqm. One of the reasons for the low uptake of the technology by most farmers could be little or no involvement of farmers in the process of developing the technology which constrains farmers’ capacity and confidence to apply the technology.

Most of the impact studies on groundnut and other legumes have been conducted in some part of Africa [6, 7, 16, 30] while there is a dearth of evidence on the actual as well as the potential impacts of improved groundnut technologies that support policy decisions in Ghana as well as West Africa. Moreover, several ex-ante impact studies show that agricultural technology adoption would have positive economic benefits to smallholder farmers in Africa [14, 31, 32]. These studies computed the magnitude of the aggregate or market level economic benefit or surplus associated with adoption of new agricultural technologies, but they did not allocate the benefits to potential market participants including poor producers and consumers. While it is difficult to make a priori generalizations about the impacts of agricultural technologies on the poor and distributional benefits are context specific [33], limited studies are available to justify that the existing efforts to promote improved agricultural technologies are pro-poor [7, 16, 34]. Therefore, this study addresses these gaps. Specifically, we estimate the potential impact of a new groundnut plant density on welfare of smallholder farmers at the household level and show the pattern of poverty reduction during the expected years of adoption. The results of our impact estimations are based on a systematic prediction of adoption of the technology which is not the case in the previous ex ante impact studies [7, 16, 34].

The results show that all planting density options are more profitable than the farmers’ practice while the highest profit is associated with the highest density (i.e., 22 plants/sqm). Prediction results show that about 62% of groundnut farmers in the study areas are expected to adopt the densest planting density. This level of adoption will reduce the incidence of extreme poverty among smallholder farmers by about 3.6% if the farmers have access to the international groundnut market and by about 2% if they do not have. The intervention will also reduce poverty gap and poverty severity.

The rest of the paper has been organized as follows. Section 2 provides a brief description of rural livelihood and groundnut production in northern Ghana. Section 3 describes the methods of the study including data sources and methods of data analysis. Section 4 presents and discusses the results while Section 5 concludes the paper and shows policy implications of the findings.

## 2. Rural livelihoods and groundnut production in northern Ghana

Farmers in northern Ghana (also known as the Savannah) depend on both crops and livestock to sustain their households. They grow different types of crops including cereals, legumes, fruits, vegetables, root crops, and tuber crops while raising different kinds of animals such as goats, sheep, cattle, pigs, and poultry [20]. The average landholding of the Savannah is 2.8 hectare [35]. While farmers use different practices and inputs, productivity is generally low because of a low level of input application [36–38]. In addition, the erratic weather condition in this part of the country imposes adverse effects on agricultural productivity. Furthermore, farmers have low access to markets and low share in the marketing margin [39]. These factors have contributed to low living standards. While poverty in Ghana is predominantly a rural phenomenon, rural Savannah is the most affected. For instance, in 2016/17, this part of the country contributed to about two-third of the total incidence of extreme poverty in the country [40].

**Fig 1:**
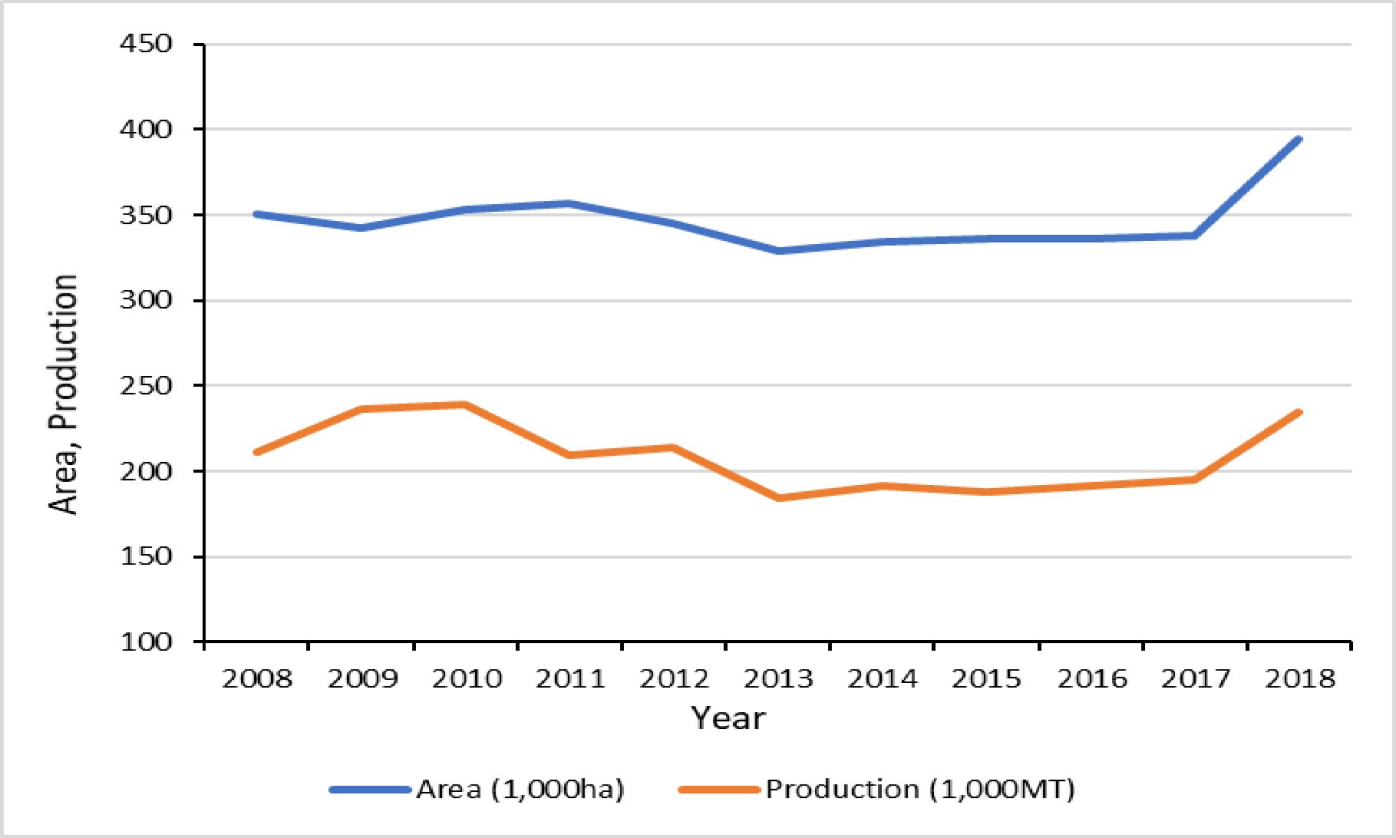
Groundnut production and area coverage for Ghana; data source [42].

The Savannah is the major groundnut producing agro-ecological zone in Ghana. It accounts for 94% of the total groundnut production in the country [39]. Farmers produce groundnut as a sole crop and, to some extent, as an intercrop with cereals. Despite its suitability for groundnut production, the Savannah is characterized by low grain yield and stagnant production (Fig 1). Grain yield with shell is estimated at 1.3t/ha which is about 37% of the potential yield [25]. One of the reasons for low productivity is the establishment of sole groundnut crops in wide rows, while studies show that higher plant density can increase yield [27, 28, 41].

**Fig 2:**
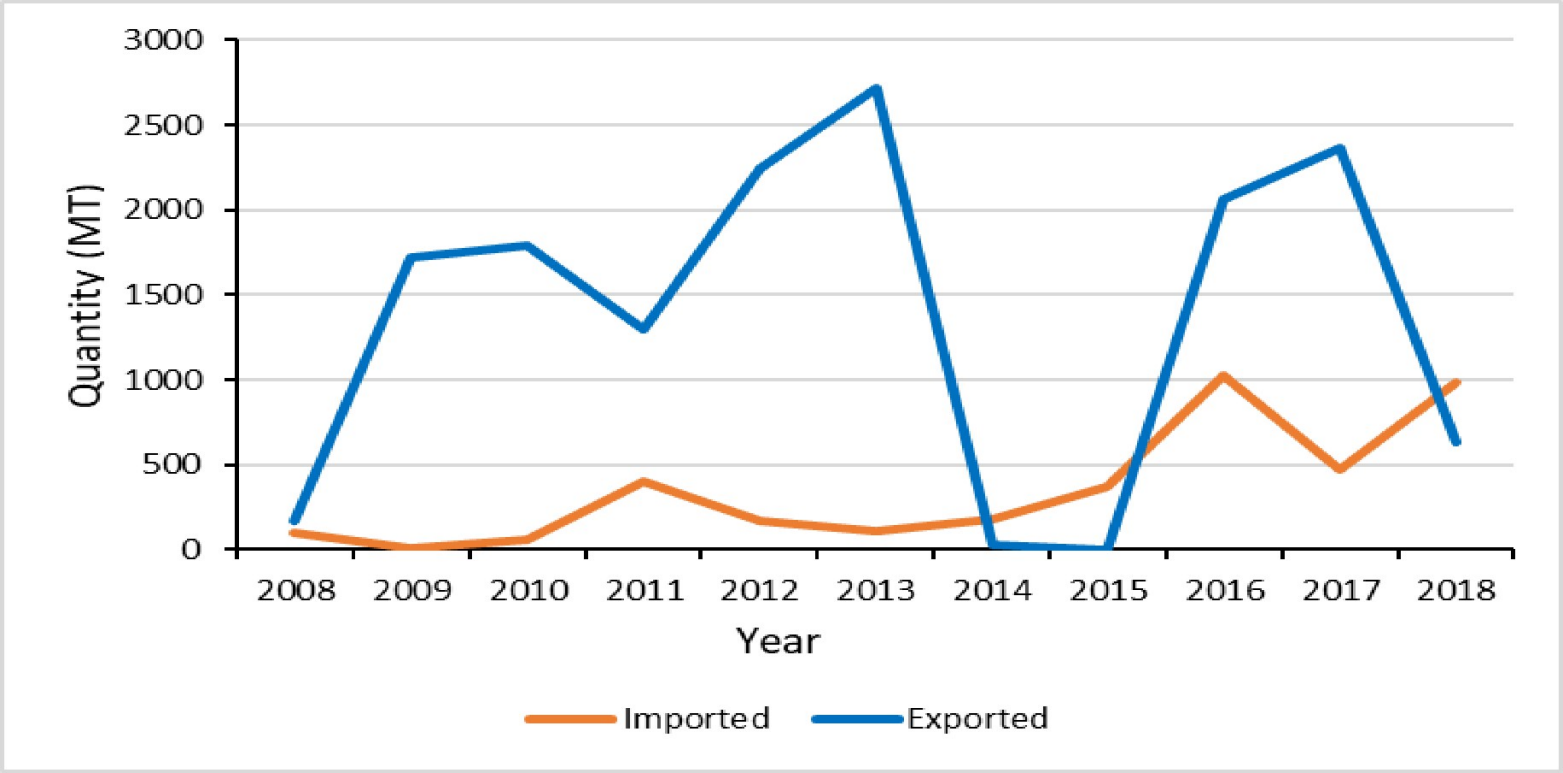
Groundnut export and import of Ghana; data source [42].

Farmers produce groundnut for home consumption and sale [39]. It is a source of protein and oil which constitutes much of the Ghanaian cuisine. It is also a source of cash for farm households as it fetches higher prices compared to cereals. Groundnut is sold both in domestic and export markets. While Ghana is a net exporter of groundnut, the volume of export is less than 1% of the total production (Figs 1 and 2). Marketing of groundnut involves different actors, including farmers, wholesalers in the Savannah, wholesalers in Accra, agents in Accra, processers in Accra, retailers in different towns, and exporters. Exporters send their products mainly to the European Union and African countries such as Senegal and South Africa [39].

## 3. Methods of the study

### 3.1. Data used

We used data from different sources, namely on-farm experiments, focus group discussions, a household survey, and secondary data. These are described separately in the following paragraphs.

**Fig 3:**
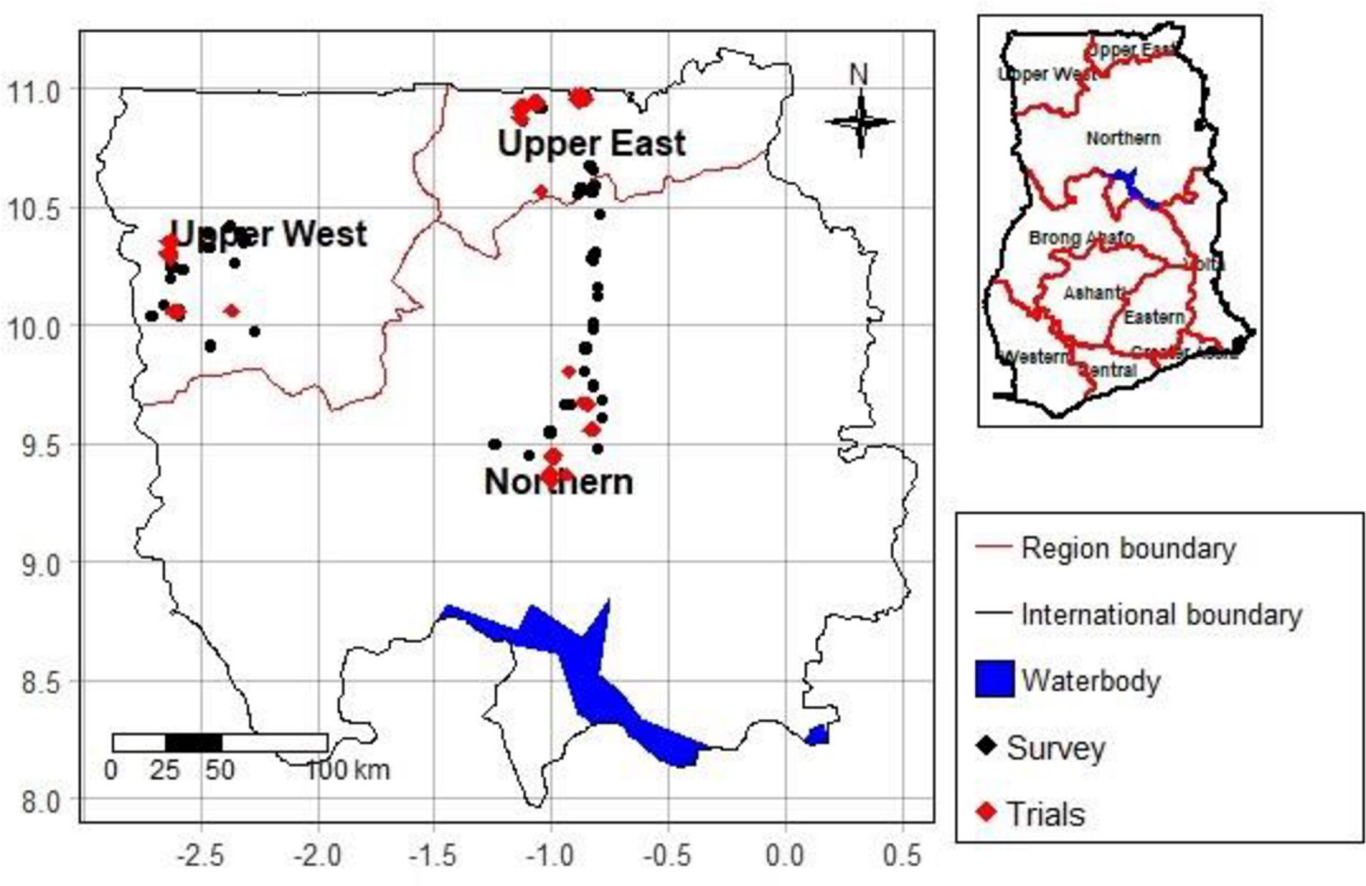
Location of trial sites and survey sites in the study areas. Source: The administrative layers were downloaded from the Global administrative boundaries database (GADM) which does not need license for academic publishing (https://gadm.org/license.html).

On-farm experiments were conducted in 2016, 2017, and 2018 to assess the effect of plant density on grain yield of groundnut in 12 intervention communities of the Africa RISING Project which are found in three regions of northern Ghana, namely, Northern Region, Upper West Region, and Upper East Region (Fig 3). An experimental site (also known as a technology park) was established in each community for farmers to observe, participate, and learn about the technology. The experiments in the “technology parks” were closely supervised by the researchers while farmers participated at every field activity. In addition, four to twelve farmers in each community established replicas of the “technology park” trials on one acre of land (also known as upscaling trials) in each year with input support from the Project so that they could closely assess the benefit of the new practice. The upscaling trials were managed by the farmers with technical guidance given by the researchers. With regards to the “technology park” trials, a factorial treatment combination of six improved groundnut varieties and four plant densities were laid in strip plot design with four replications per region. The six improved groundnut varieties were: Chinese, Yenyawoso, Samnut 23, Azivivi, Mani pinta and Samnut 22 [see also 43]. However, as this study focuses on the impact of increasing plant density on grain yield, we considered the data associated with the variety which farmers commonly use (the Chinese variety). The four plant densities include: 22, 15, 11 and 9 plants/sqm. The 9 plants/sqm density was used as a control representing farmers’ practice [26]. To achieve the above plant densities, the groundnut seeds were sown one seed per hill at spacing of 30 x 15, 45 x 15 60 x 15 and 75 x 15 cm2 for the plant densities 22, 15, 11 and 9 plants/sqm respectively. The 22 plants/sqm density was considered as a maximum value of our treatments since plant densities above this did not result in significant increase in pod or seed yield as shown by previous studies in Ghana [28, 29]. Weeding was done manually at three weeks after planting for all the plots and before initiation of pegs in the 15, 11 and 9 plant/m2 plots in line with good agronomic practices. No fertilizer was used in the trials. The grain yield was measured from the two middle rows of each treatment plot to reduce edge plant rows effect by harvesting the pods of groundnuts from the two middle rows, oven dried at 65°C to a moisture content of 12% and cracked to weigh the seeds as grain yield. We used the data to conduct cost-benefit analysis and to estimate the market level effect of the new technology (see Sections 3.2.1 and 3.2.3).

We conducted 22 focused group discussions (FGDs) in 11 communities of the study areas with farmers who hosted and participated in the trial of the new practice. A total of 189 people (90 men and 99 women) participated in the FGDs with average participation of eight people per FGD. Men and women were considered in the FGDs to accommodate the diversity of perceptions in the farming community. The two gender-based groups were interviewed separately to avoid possible suppression of women voices when interviewed together with men. We used the questions and response options embedded in Adoption and Diffusion Outcome Prediction Tool (ADOPT) Smallholder version 1.0 in the FGDs [44]. While the list of factors influencing adoption can be long, ADOPT considers 22 variables based on the review of literature on adoption (e.g. [45–48]) (see Table A1 in the S1 File for full list of the variables). Most of the variables were measured along the 1-to-5 scale, 1 indicating the least desirable characteristics of the innovation or the population to adopt the technology and 5 indicating the most desirable one. However, some variables were measured along the -3 (least desirable) to +4 (most desirable) scale. The positive (negative) values in the latter scale show the advantage (disadvantage) of the technology as compared to the farmers’ practice. We used several steps to complete data collection. First, farmers provided their private scores for each variable considering the new practice. Second, we computed the mean values for each variable for each group discussion. Third, we made discussions with the farmers in groups to validate/adjust the mean values. Fourth, we computed the mean value for each variable by aggregating the results of all focus group discussions. Fifth, we discussed with scientists from International Institute of Tropical Agriculture (IITA), Savannah Agricultural Research Institute (SARI), and International Center for Research In Semi-Arid Tropics (ICRISAT) on the results of farmers’ assessments to seek validation on some attributes which demand scientific opinion (such as externalities associated with the technology as they may affect future profitability of the farm business and the risk associated with the technology). The data collected through this process were used to predict aggregate adoption as explained in Section 3.2.2.

We also used data from a household survey conducted by the International Food Policy Research Institute (IFPRI) in 2014. The survey was implemented to establish a baseline for a research-for-development project known as “Africa Research In Sustainable Intensification for the Next Generation” (Africa RISING). The survey covered 1284 farm households located in the three regions mentioned above (Fig 3) and collected data on diverse topics including household demographic characteristics, land and other farm resources, crop and livestock production, off-farm employment and income, access to institutional services, access to market, and household expenditures. A detailed description of the sampling procedure and the data can be found in [49]. We used the survey data to estimate the welfare effect of the new practice at household level (see Section 3.2.4).

The data which involved human subjects were collected following institutional ethical standards. The household survey was approved by the institutional review board (IRB) of International Food Policy Research Institute. A clear explanation was given to the participants on the objectives of the survey and all of them were asked for their verbal informed consent to willingly participate in the study. Participants were informed that they could skip any question they did not want to answer and even could withdraw at any time from the interview. The protocols of the on-farm experiment and the discussions made with the host farmers were reviewed and approved by the Africa RISING Program coordination team. The data were analyzed anonymously; farmers’ names had been removed before the data were uploaded to an online repository.

### 3.2. Data analysis

#### 3.2.1. Cost-benefit analysis

We computed gross margin based on grain yield, grain price, seed cost, labor cost, and draft power cost. Data on grain yield were generated through the on-farm experiments. Labor cost (associated with various farm activities) and draft power cost were based on discussions made with key informants and the on-farm experiments. Data on cost of seeds were collected from Seed Producers Association Ghana (SEEDPAG) for the respective regions. Data on market grain prices (for the years 2016–2018) were collected from a company engaged in collection of price data for agricultural products in Ghana (http://www.esoko.com). Based on [50], the market grain prices were adjusted downwards by 33% accounting for market transaction costs to arrive at farm gate prices.

#### 3.2.2. Adoption prediction

**Fig 4:**
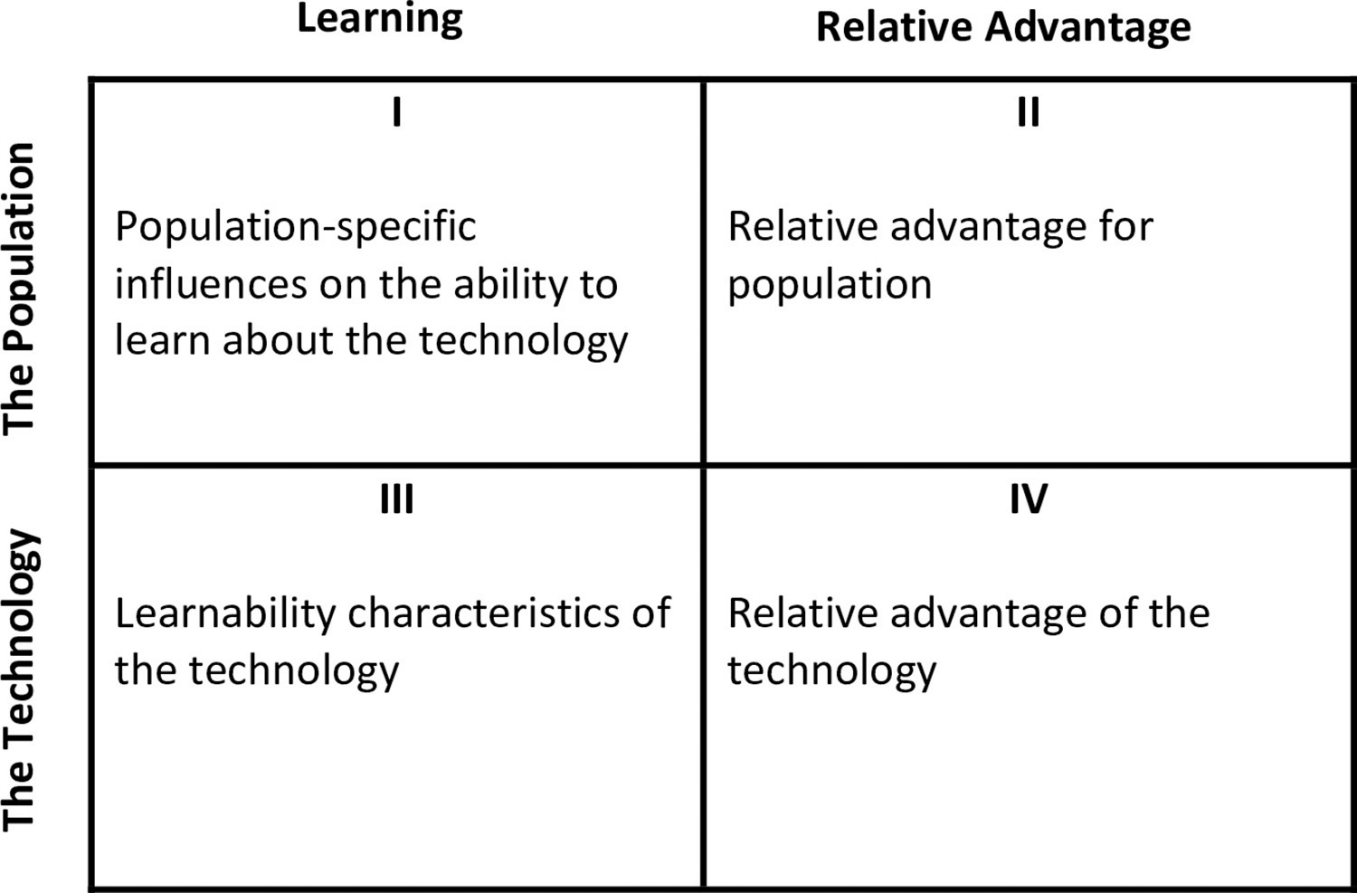
The four vectors of factors influencing adoption of new agricultural technologies; source [44].

We used the framework proposed by [44] to predict the adoption of the new groundnut technology. According to the framework, there are two overarching factors which affect adoption decisions in agriculture namely, the relative advantage of a technology and the learning process. The relative advantage of a technology determines the peak adoption rate while the learning process determines the time required to reach the peak adoption rate. For example, profitability of a technology (relative to the existing technologies) may affect the maximum number of farmers who will adopt a technology while access to extension service may affect the time required to reach the maximum number of adopters. The two overarching variables can be characterized by population-related features and technology-related features. Combining the two pairs of factors (relative advantage vs learning process and population-related features vs technology-related features) would result in four distinct vectors of factors influencing adoption (Fig 4). Factors in the second and the fourth quadrants of Fig 4 influence the peak adoption rate whereas those in the first and the third quadrants influence the length of time to reach the peak adoption rate.

**Fig 5:**
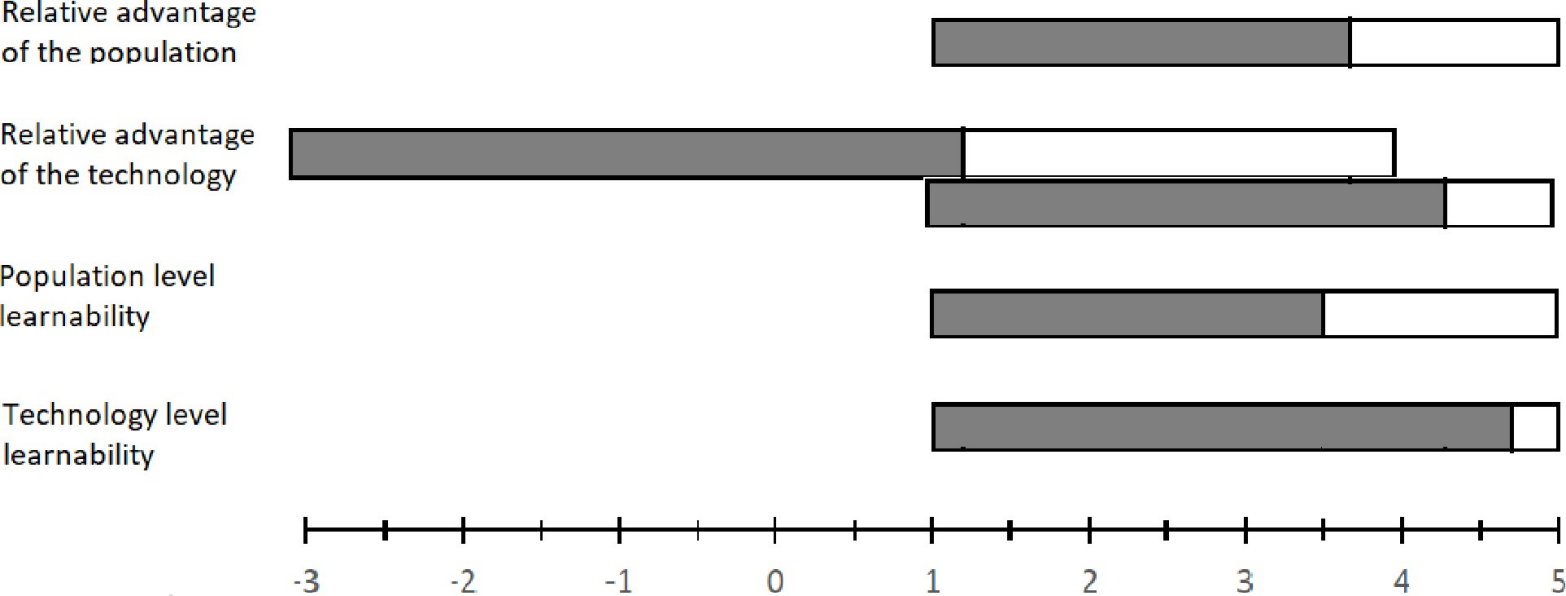
Farmers’ perception scores of the new groundnut plant spacing by four vectors of adoption factors. Legend: The right tips of the dark bars indicate the mean values of farmers’ perception scores of the vectors of the adoption factors.

Fig 5 displays the summary of the results of the scores corresponding to the four categories of the variables based on the data collected through FGDs and expert consultations. It reveals that the adoptability of the technology is generally high in the area. The scores corresponding to those variables which were measured using the 1 to 5 scale were at least 70% close to the maximum value. However, the relative advantage of the technology was perceived by the farmers to be moderate in terms of those variables (e.g., benefit to community members beyond the adopters and contribution to risk exposure) which are measured using the -3 to +4 scale. We conducted the adoption prediction by selecting an appropriate score for each of the 22 variables embedded in the ADOPT Smallholder Version 1.0. We conducted sensitivity analysis by moving one step up and down along the measurement scale of each variable (see Table A1 Column E in S1 File for the reference scores for base scenario predictions).

#### 3.2.3. Economic surplus analysis

We used the economic surplus model (ESM) to estimate the market level effects of the new technology. The ESM is a partial equilibrium model which is commonly used to estimate the benefits and costs of technological changes [7, 16, 34]. The most important step in the ESM analysis is to calculate the supply shift parameter (K_t_) which measures the per unit cost reduction that may occur due to the introduction of a new productivity enhancing technology. The supply shift can be computed using the following equation [17].


$$K_{t}=\left[\frac{E(Y)}{\varepsilon }-\frac{E(C)}{1+E(Y)}\right]\rho A_{t}$$ (1)

Where E(Y) is the expected proportionate yield change per hectare if the new groundnut technology is adopted, ε is the elasticity of supply of groundnut, E(C) is the proportionate change in input cost per hectare (if any), ρ is the probability of success of the new technology, A_t_ is the rate of adoption in year t.

We derived the estimates of E(Y) and E(C) from the costs benefit analysis and that of A_t_ from the adoption prediction model. We assumed ρ = 1 since the target technology has completed its trial stage and has been approved by the scientists for scaling. We couldn’t find supply elasticity of groundnut for Ghana. [17] suggest that assuming a supply elasticity of 1 is a good starting point in the absence of accurate information since the supply elasticities of most of the commodities are close to 1 in the short and medium terms. Based on this theoretical suggestion and following [16], we assumed ε = 1 in our analysis. We also assumed demand elasticity (γ) equals to -0.5 based on [16].

The theoretical explanation behind Eq 1 is that the productivity changes arising from the introduction of the new groundnut practice will shift the market supply of groundnut to the right, which in turn will change the equilibrium market price. Producers as well as consumers may benefit from such changes depending upon the market context. In a competitive market context, the share of the benefit (the surplus) between the producers and consumers is determined by three critical factors, namely price elasticity of demand, price elasticity of supply, and the extent of the supply shift. The total economic surplus (Δ*ES*) attributed to the technological change is the sum of consumers’ surplus (Δ*CS*) and producers’ surplus (Δ*PS*). Following [17], this can be written mathematically as:

$$\Delta ES=\Delta CS+\Delta PS=P_{0}Q_{0}Z_{t}(1+0.5Z_{t}\gamma )+(K_{t}-Z_{t})P_{0}Q_{0}(1+0.5Z_{t}\gamma )=K_{t}P_{0}Q_{0}(1+0.5Z_{t}\gamma )$$ (2)

Where P_0_ and Q_0_ are the prices of the commodity and the quantity supplied before the adoption of the new technology, Z_t_ is the percentage reduction in market price because of supply shift determined by the extent of reduction in per-unit cost of production arising from technological change (*K*_*t*_), price elasticity of supply (ε), and the price elasticity of demand (*γ*) such that:

$$Z_{t}=K_{t}\varepsilon /(\varepsilon +\gamma )$$ (3)

We conducted our analysis under two alternative assumptions on the market situation. First, we assessed the impacts of the technology considering Ghana as a small groundnut producer that has access to the international market for its groundnut produce. This assumption is plausible because Ghana is a net groundnut exporter [39]. We also assumed stable international prices. In a small open economy, the entire surplus goes to producers because a small economy cannot affect the international market price. Hence, domestic consumers will not enjoy the benefit of lower prices arising from the introduction of the new technology. Thus, Eq 2 will reduce to:

$$\Delta ES=\Delta PS=K_{t}P_{0}Q_{0}(1+0.5Z_{t}\gamma )$$ (4)

Second, we conducted our analysis under a closed economy assumption. This assumption also makes sense because not every product can enter the international market even if the exporting country follows an export-oriented policy. Significant volumes of exports from developing countries fail to enter the international market as they do not fulfill the quality standards of the importing countries. For instance, the EU imposed restrictions on groundnut imports from Ghana between 2009 and 2013 because of aflatoxin contamination exceeding the tolerated level in the EU. As a result, the Ghana Export Promotion Agency (GEPA) conducted a series of training to farmers to increase the quality to the level required by the EU. Moreover, there may be heterogeneity of accessibility to export markets across the population which makes the closed economy assumption plausible at least in the short run. In a closed economy, the total economic surplus associated with technological change will be shared between producers and consumers: adopter producers will benefit because of a reduction in per-unit costs of production while domestic consumers will benefit from a reduction in product prices associated with the supply shift. However, non-adopter producers will be adversely affected because of the price reduction associated with adoption.

In this case, the change in producers’ surplus is:

$$\Delta PS=(K_{t}{-Z_{t})P}_{0}Q_{0}(1+0.5Z_{t}\gamma )$$ (5)

While the change in consumers surplus is:

$$\Delta CS=P_{0}Q_{0}Z_{t}(1+0.5Z_{t}\gamma )$$ (6)

#### 3.2.4. Estimating the welfare effect at household level

The economic surplus model provides the potential impact at market level but does not show how much those households who adopt the new groundnut technology would benefit. However, the welfare effect of the technology is of interest to development practitioners, policy makers, and the farm households. Therefore, allocating the surplus to households who are likely to adopt the technology and estimating the impact on poverty would be the next task. We followed several steps to complete this task. First, applying the logistic regression model, we predicted the probability that each of the households would adopt the new technology and ranked the households in descending order based on the predicted probabilities. The assumption is that those households who are associated with larger probability values in the logistic model will adopt the technology ahead in time while those who are associated with smaller probability values will take more time to adopt. This basic assumption links this household-level model with the aggregate adoption prediction of the ADOPT which involved a broader range of factors. Since scaling of the technology has just started and adoption data was not available for the new groundnut technology, we used the application of other improved technologies (such as improved varieties, fertilizers, and pesticides) on groundnut as a proxy variable. This means that households who have the experience of applying improved technologies on groundnut are assumed to have high propensity to adopt the new practice. A similar approach has been used in previous studies to estimate the potential impact of new agricultural technologies [7, 16].

Second, we computed the household level welfare measure from the household survey data and compared it with the national poverty line. We used annual food expenditure per adult equivalent (hereinafter income per capita) as a proxy of household welfare. We extrapolated the annual food expenditure from households’ food expenditure within seven days before the survey time. We computed adult equivalent based on household demographic variables (age and sex) and conversion factors suggested by [51]. We used a daily food expenditure of 3.53Ghc per adult equivalent as poverty line based on [40] after adjusting for inflation. This expenditure threshold for extreme poverty is equivalent to the new international poverty line (i.e., $1.9/day/person) which was introduced by The World Bank in 2016. The change in the real per capita GDP of Ghana was almost zero between 2014 (the survey year) and 2019 (the base year for our impact analysis) [52]. Therefore, we assumed that the per capita income of the sample farmers remained unchanged between this period.

Third, we estimated the pre-adoption aggregate poverty level (P_α_) for the whole sample of households in the survey data using the Foster-Greer-Thorbeck (FGT) formula as specified below.


$$P_{\alpha }=\frac{1}{N}\sum_{t=1}^{q}{\left[\frac{z-y_{i}}{z}\right]}^{\alpha }$$ (7)

Where *N* is the total number of households in the sample, *q* is the number of poor households in the sample, *y*_*i*_ is the household per capita income, z is the national poverty line, and *α* is a parameter of inequality aversion. Please note that when *α* = 0, the FGT becomes the headcount poverty index; when *α* = 1, it becomes the poverty gap index and measures the aggregate poverty deficit relative to poverty line; when *α*>1, it reflects increased sensitivity to inequality among the poor.

Finally, we estimated the post-adoption income of each household by adding the changes in economic surpluses and reduction in output prices (analyzed based on Eqs 3–6) to the pre-adoption income. We used household level pre-adoption groundnut production to allocate the economic surplus among producers. We assumed that every area of groundnut would be planted to groundnut at the new spacing once a farmer adopts it given that this is a relatively low-cost and less-complex technology. We also assumed that existing groundnut growers would continue to grow the crop, but new growers would not come in. For consumers, we allocated the economic surplus based on their pre-adoption annual groundnut consumption. We could do the allocation for farmer consumers only since all households covered by the survey were farmers. Since we considered impact on extreme poverty which is associated with food as defined in [40] for Ghana and that households may use the additional income from the new groundnut technology for non-food expenses, we adjusted the total change in income downwards based on the share of food expenditure in households’ total expenditure. Thereafter, we re-estimated the FGT indices using the post-adoption income and compared with the pre-adoption income to arrive at the potential impact on poverty that would result from the adoption of the technology. We used the prediction results in Section 3.2.2 to estimate the expected household welfare changes in all years during the adoption period.

## 4. Results and discussion

### 4.1. Results of cost-benefit analysis

The results of the partial budget analysis show that all spacing options are profitable, with profit increasing from the least dense spacing (i.e., the farmers’ practice) to the densest spacing (Table 1). The benedict-cost-ratio associated with all new spacing practices are substantially greater than 1 implying positive financial return to investment while the one associated with the farmers’ practice is close to 1 implying that farmers could only operate at breakeven point. The 22 plant/sqm density provides about 87% profit for every Ghana cedi invested, which is the highest return. The cost of production shows a moderate increase as planting density increases. However, the cost per kilogram of groundnut grain produced decreases as planting density increases implying that increment in the gross financial return outweighs the increment in the cost of production. The lowest cost is 1.6Ghc per kilogram of grain produced, which shows a 44% cost reduction as compared to farmers’ practice. In subsequent analysis, we focus on the 22 plants/sqm density as it is the most optimal.

**Table 1:** Partial budget analysis of on-farm groundnut spacing trials.

	22 plants/sqm	15 plants/sqm	11 plants/sqm	9 plants/sqm
Average groundnut yield (kg/ha)	966	729	621	492
Average farmgate price of groundnut grain (Ghc/kg)	3	3	3	3
Gross benefits (Ghc/ha)	2898	2187	1863	1476
Total variable cost (Ghc/ha)	1548	1476	1447	1408
Gross margin (Ghc/ha)	1311	663	406	57
Benefit-Cost Ratio	1.87	1.48	1.29	1.05
Unit cost of grain production (Ghc/kg grain produced)	1.6	2.02	2.33	2.86
% change in yield compared to farmers practice	96	48	26	
% change in total variable cost relative to farmer practice	10	5	3	

### 4.2. Predicted aggregate adoption

**Fig 6:**
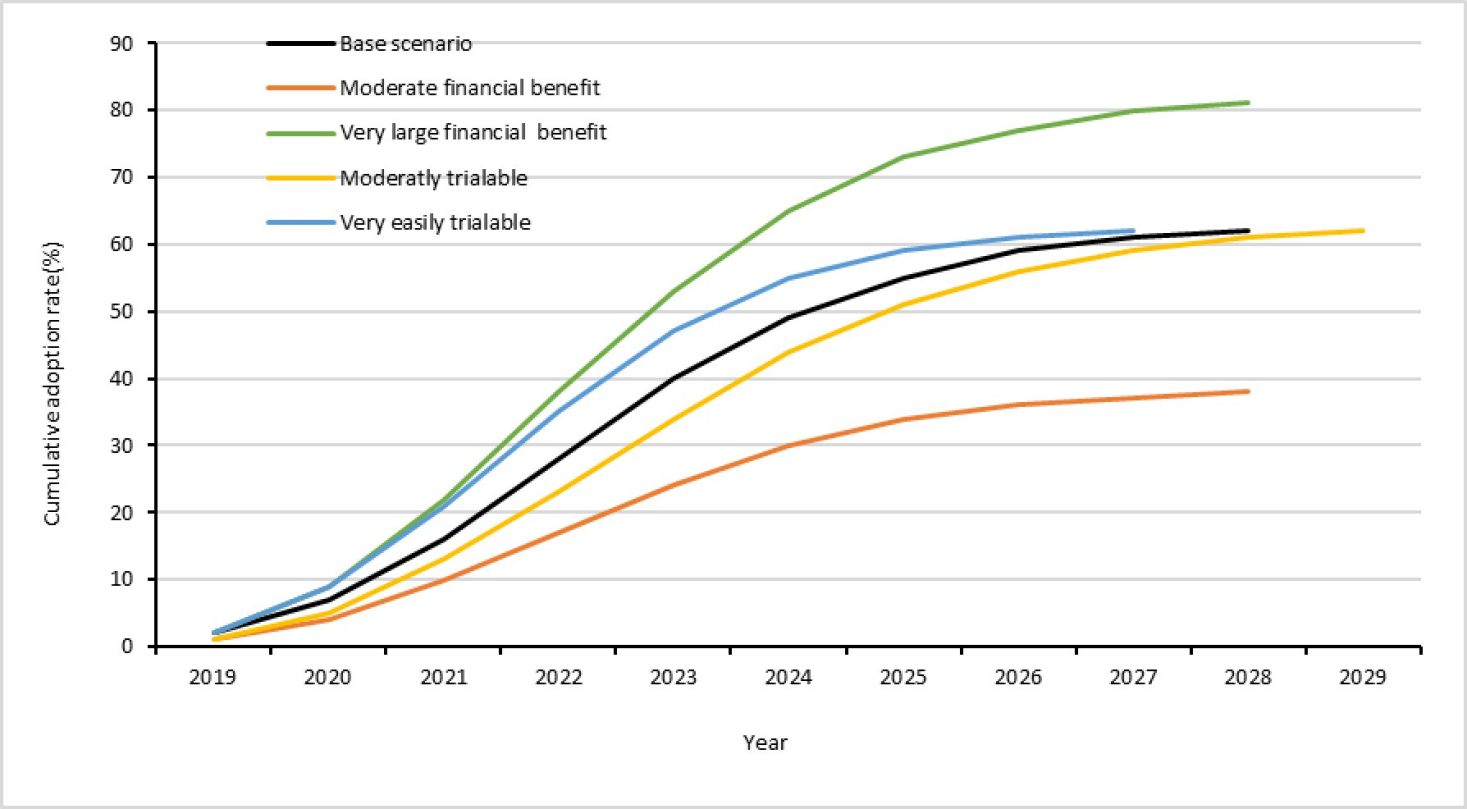
Predicted adoption and its sensitivity to farmers’ perceptions on financial benefit and trialability of the new groundnut plant spacing.

Fig 6 shows the adoption predictions based on ADOPT. The year 2019 was used as a base year in the prediction because the technology completed its experimental phase in 2018 and got approval by the scientists for out-scaling. The result shows that the peak adoption rate of the most optimal spacing is about 62% for the base scenario as displayed by the black line. The peak adoption rate requires about nine years to achieve counting from the base year of the prediction i.e., 2019 in our case. Adoption predictions made elsewhere using a similar tool indicate a bit slower speed of adoption than ours [53, 54]. [53] found that adoption of water harvesting technologies in Jordan would reach peak adoption after 12.4 years. Similarly, [54] predicted that adoption of fertilizer in central Tanzania would reach peak level after 12.8 years while this time would be extended by about two years if tied-ridges were included in the technology package. The faster speed of adoption predicted in our case could be related to the fact that the technology we considered is a low-cost technology with little demand for purchase of commercial inputs and hence can be tried even under severe financial constraints. Moreover, as perceived by the farmers, a majority of the farmers do not need a new skill to apply the technology as it is not complex. Many ex-post adoption studies on legumes focused on factors affecting adoption and did not report actual adoption rates [e.g. 55, 56]. A study conducted in Uganda showed that adoption of high yielding groundnut varieties was about 63% [57].

The base scenario prediction was made based on the perception of the farmers who hosted and managed the on-farm trials (see Table A1, Column E in S1 File for reference values). However, these farmers were not randomly selected and hence their perception may not represent the whole population of smallholder groundnut producers in their communities. Therefore, we conducted sensitivity analysis of the adoption prediction with respect to the variables which showed most sensitivity. The peak adoption rate was the most sensitive to the relative financial benefit of the technology while the speed of adoption was the most sensitive to the trialability of the technology. Fig 6 also shows the temporal adoption patters when the values of these two variables increase or decrease by one step relative to the values of the base scenario along the measurement scale. The peak adoption rate will reach 81% when very large financial benefit is considered (as shown by the green line) while it will reach 38% if moderate financial benefit is considered (as shown by the red line). The peak adoption rate can be realized within eight years if farmers can try the new technology very easily (as shown by the blue line) whereas it may take ten years if they can do it with moderate difficulty (as shown by the yellow line). The peak adoption rate and the time required to reach it are also sensitive to other factors including the impact of the technology on the risk exposure of farmers, its impact on the ease and convenience of existing agricultural practices, relative upfront cost of the technology, and productivity/profit orientation of farmers (see Fig A1 in the S1 File). The other factors effecting the speed of adoption include short term financial constraints of the farmers, complexity of the technology to apply, and the existence of knowledge and skill relevant to the application of the technology (see Fig A2 in the S1 File).

### 4.3. Predicted household level adoption

Predicting household level adoption probabilities was an important step to estimate the impact of the new technology on the welfare of smallholder farmers. We applied logistic regression on a household survey data to predict household level probabilities considering several factors. Table 2 shows that several factors affect the probability of adoption of the new groundnut technology at household level (see Table A2 in the S1 File for descriptive results). Area of land allocated to groundnut positively affects the adoption of the new technology. The larger the farm area a farmer allocates to groundnut the higher the probability that the farmer would adopt the new groundnut technology. Distance of plot from home has a negative effect on adoption which means farmers are more likely to apply new technologies to groundnut plots close to their home than the distant ones. The result supports the findings of some other studies [38, 58]. Other factors enhancing adoption include area under major cereals, off-farm activity, and participation of farmers in social groups. The positive effect of major cereals and off-farm activity on the adoption of ground technology could show that farmers use income from other sources to finance groundnut production. The positive effect of farmers’ participation in other social organizations could be because of the instrumentality of these organizations to exchange information about new agricultural practices. The results also show that there are regional differences in the expected adoption. Farmers in the Upper West and the Northern regions have lower probability of adoption of the new groundnut technology than those in the Upper East Region. The higher probability of adoption in the Upper East Region could be related to its high population density which, in line with the Boserupian hypothesis, may have induced demand for land intensification [10, 59, 60].

**Table 2:** Logistic model estimates of factors affecting adoption of improved groundnut practices.

	Description	Coefficients	Marginal effects
Sex of household head	Dummy variable for sex of household head, 1 for male and 0 for female	-0.152 (0.303)	-0.027 (0.054)
Age of household head	Age of household head in years	-0.004 (0.008)	-0.001 (0.001)
Education of household head	Dummy variable, 1 for literate and 0 otherwise	-0.014 (0.264)	-0.003 (0.047)
Household size	Number of people dwelling in the same household	0.022 (0.022)	0.004 (0.004)
Area under major cereals	Area under major cereals (maize, rice, and millet) (ha)	0.077** (0.038)	0.014** (0.007)
Area under groundnut	Area under groundnut production (ha)	0.372*** (0.140)	0.067*** (0.025)
Farmland location	Dummy variable for location of farmland under groundnut production, 1 if located at homestead and 0 otherwise	-1.091*** (0.401)	-0.196*** (0.070)
Number of plots	Number of plots the household cultivated	-0.038 (0.079)	-0.007 (0.014)
Livestock	Livestock ownership measured in TLU	0.006 (0.018)	0.001 (0.003)
Off-farm activity	Dummy variable for participation in off-farm activities, 1 if household member participated in off-farm activities and 0 otherwise	0.419* (0.237)	0.075* (0.042)
Credit access	Dummy variable for access to credit, 1 if household received input loan and 0 otherwise	0.198 (0.381)	0.035 (0.068)
Market access	Distance of community from nearest market town	-0.009 (0.008)	-0.002 (0.001)
Membership in social organization	Dummy variable for household membership in farmer-based organization, 1 if household participated and 0 otherwise	0.491** (0.243)	0.088** (0.043)
Information access	Dummy variable for access to advises on agricultural production from extension, research, model farmers, and NGOs; 1 if household had access and 0 otherwise	-0.328 (0.207)	-0.059 (0.037)
Northern Region	Dummy variable for Northern Region, 1 if household is in the Northern region and 0 otherwise	-1.178*** (0.301)	-0.211*** (0.051)
Upper West Region	Dummy variable for Upper East Region, 1 if household located in the Upper East Region and 0 otherwise	-1.759*** (0.343)	-0.316*** (0.056)
Constant		-0.995 (0.789)	
Chi-sq		75.823***	
Sample size		537	

*,**,***Significant at 10%, 5%, and 1% levels of significant; Figures in parenthesis are standard errors.

### 4.4. Welfare effects of adopting the new groundnut plant density

**Fig 7:**
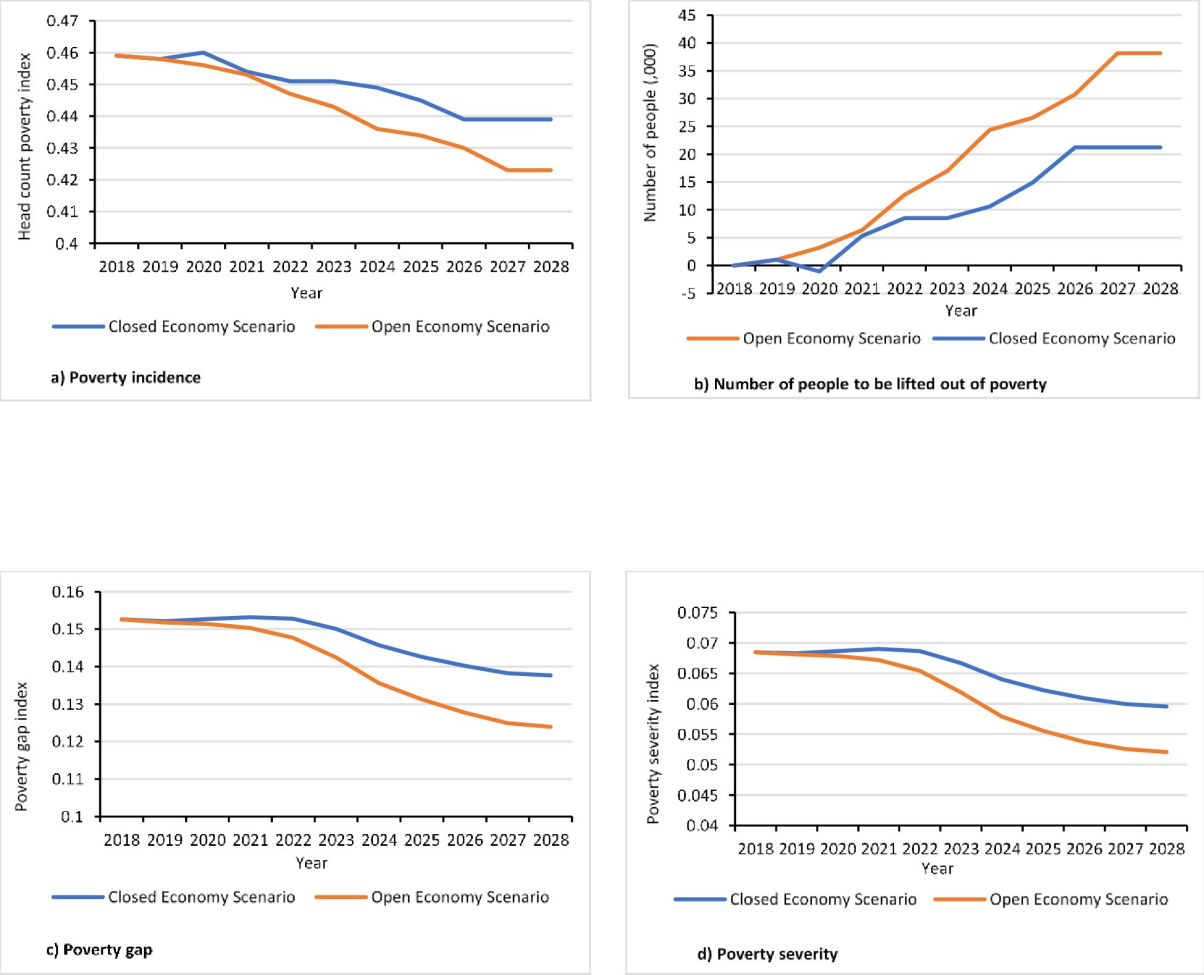
Impact of adoption of the new groundnut plant spacing on poverty.

Using the results described in Sections 4.1 to 4.3, we estimated the impact of the new groundnut planting density on poverty at household level. The results of our analysis show that the introduction of the 22 plants/sqm planting density can reduce the incidence, the gap, and the severity of poverty among smallholder groundnut producers in the study areas (see Table A3 in S1 File for associated income changes). Poverty incidence is expected to decline by about 0.36 percentage point every year until the peak adoption rate for the base adoption prediction scenario (Fig 7A). This means that by the year of peak adoption (i.e., 2028) about 3.6% of poor groundnut producers in the Savannah agro-ecology will be lifted out of poverty. This percentage corresponds to about 38,000 poor people who directly depend on groundnut in this agro-ecological zone (Fig 7B). We also analyzed the impact of the new groundnut practice on poverty gap and poverty severity among the poor (Fig 7C and 7D). Results show that adopting the 22 plants/sqm density will reduce the mean proportionate poverty gap by about 20%. This is equivalent to an additional income of about US$20 per person per annum using the Purchasing Power Parity exchange rate, which was about 1.877 [61]. The severity of poverty will decline by about 13% which also means that inequality among the poor will be reduced. These results support the findings of earlier studies on the welfare impact of new technologies for legume production [6, 7, 16]. For instance, [7] estimated that poverty incidence would decline by about 7% if all farmers adopt maize-soybean rotation in Zambia; the corresponding reduction in poverty gap and poverty severity are 21% and 27%, respectively. Similarly, [16] found that adoption of a new groundnut variety in Uganda can reduce poverty incidence by 3%, poverty gap by 8%, and poverty severity by 9.5% among the producers.

The effects of adoption on welfare will be more complicated if a closed economy is assumed. In this case, adoption is expected to reduce not only the per-unit cost of production but also the market prices. Thus, unlike the open economy the case, it can directly affect adopters, non-adopter groundnut producers, and groundnut consumers at large. Our results show that adoption would reduce poverty among adopters thereby increasing their income. It improves the welfare of non-groundnut producing consumers as it enhances their purchasing power by reducing output price levels. The incidence of poverty is expected to decline by 2% (Fig 7A). Similarly, poverty gap and poverty severity are expected to decline by about 10% and 13%, respectively (Fig 7C and 7D). Poverty gap and severity are expected to decline slowly during the initial years until 2022 but the speed of reduction will increase thereafter. This means that as adoption increases in the course of time it will be likely that a greater number of poor households will benefit from the technology, which in turn will have a positive impact on the overall rate of reduction in terms of poverty gap and severity. These results show that the impact of adoption on poverty under the closed economy is not as much as the situation under the open economy. A similar pattern was observed by [16].

### 4.5. Sensitivity analysis of the impact estimation

**Fig 8:**
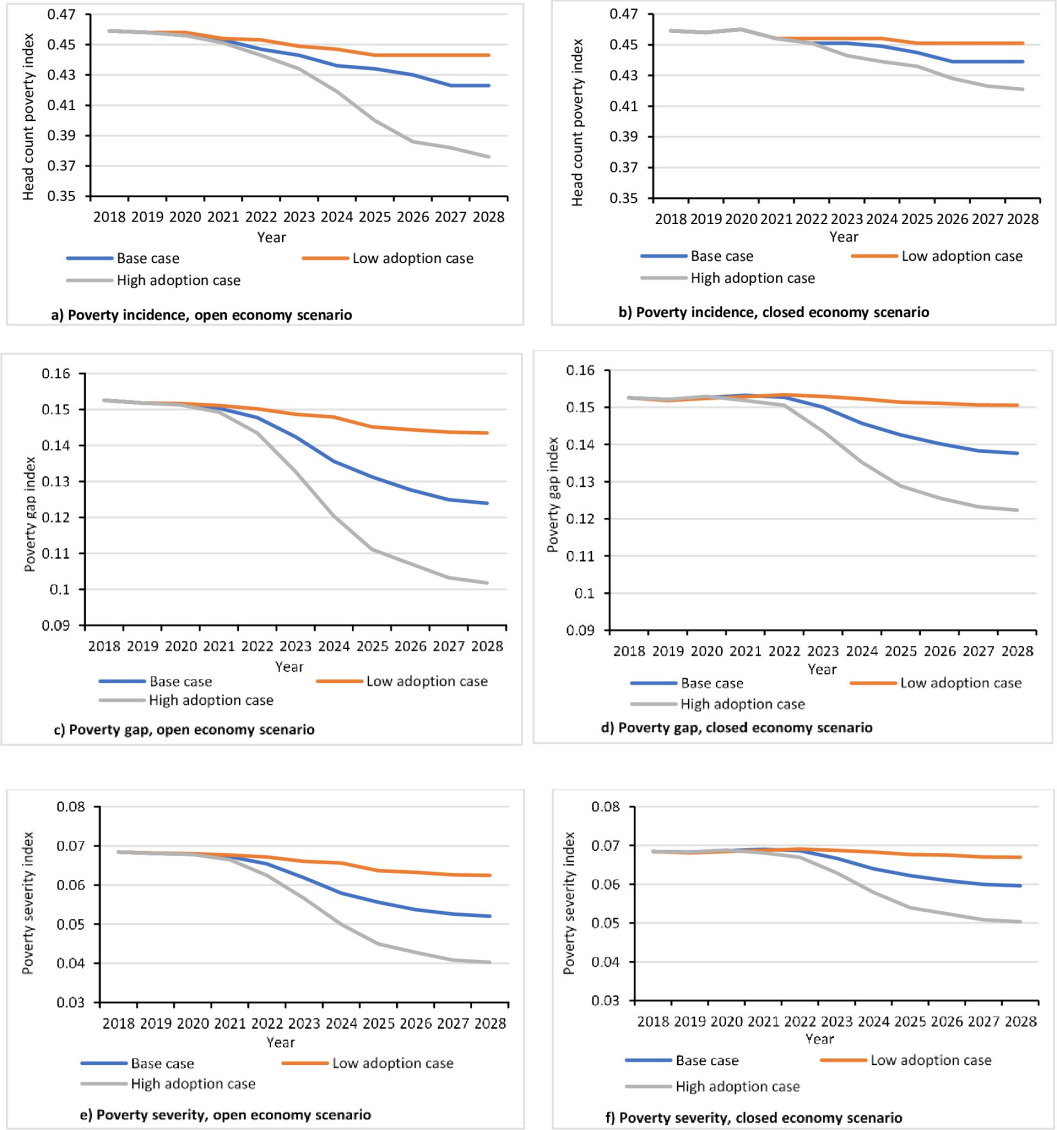
Sensitivity of poverty indices to changes in adoption rates.

Results of welfare impact analysis are highly sensitive to the expected peak adoption rate. Poverty incidence decreases by 8.3% under the open economy scenario and by 3.8% under the closed economy scenario when high peak adoption is assumed (Fig 8A and 8B). On the contrary, incidence of poverty decreases by 1.6% under the open economy scenario and by 0.8% under the closed economy scenario when low adoption rate is assumed. Similarly, the results associated with the poverty gap and poverty severity indices show that the impacts in the case of high (low) adoption assumption are notably higher (lower) than the base adoption context (Fig 8C–8F).

**Fig 9:**
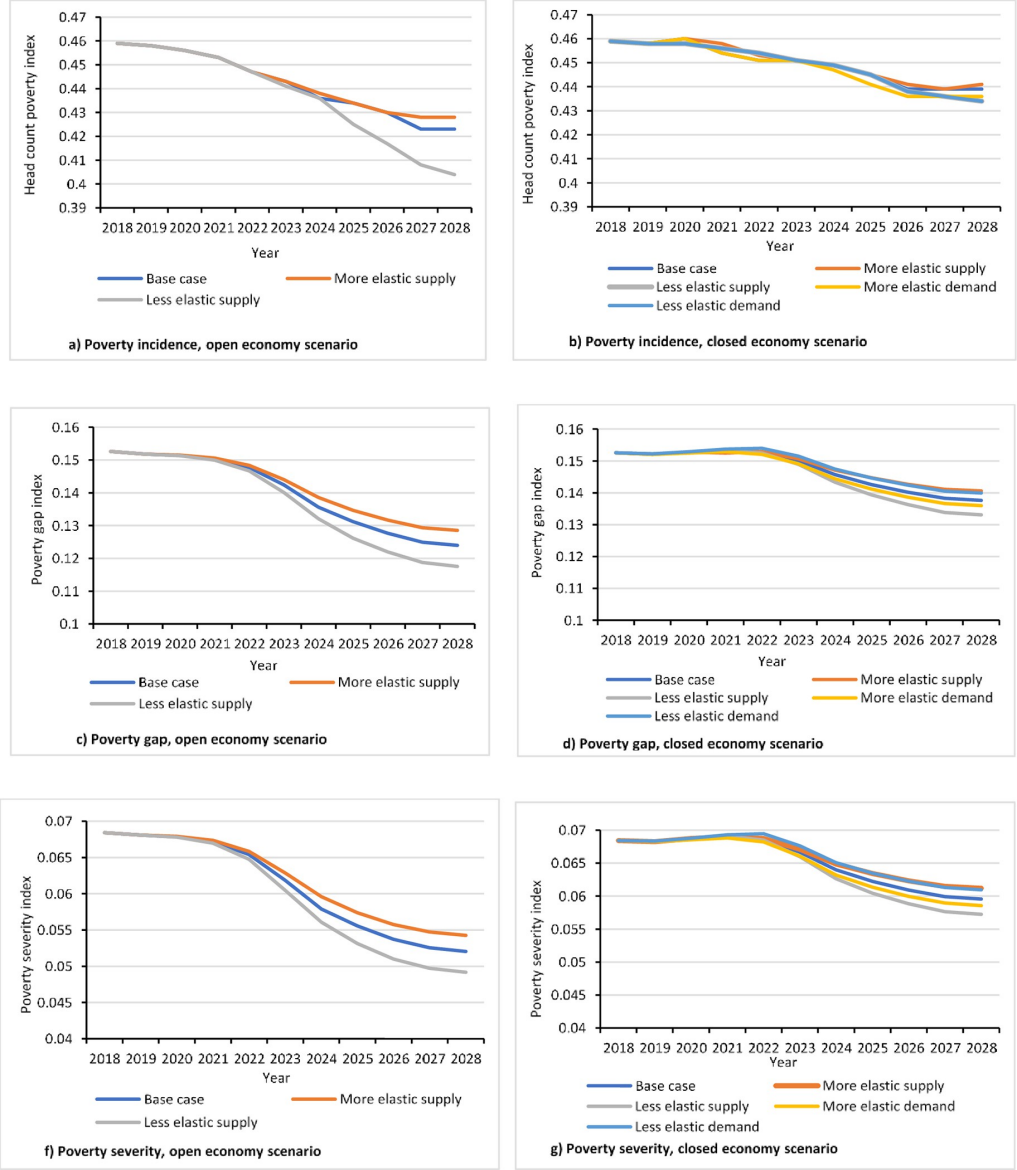
Sensitivity of poverty indices to changes in supply and demand elasticities.

We also analyzed the data by changing the values of supply and demand elasticities upward and downward by 0.2. Results show that the impacts are generally not too sensitive to changes in the elasticities within the range we considered. However, the level and pattern of sensitivity vary between the two market conditions. In the open economy case, the impact on poverty incidence is not sensitive to changes in the elasticity of supply for the first six years of the prediction period but, thereafter, the results become increasingly sensitive until the year of peak adoption (Fig 9A). In the closed economy case, the sensitivity is too small while the pattern is, by and large, uniform along the prediction period (Fig 9B). Poverty gap and poverty severity indices show low sensitivity during the first half of the prediction period while they become more sensitive afterwards (Fig 9C–9F).

## 5. Summary and policy implications

In this study, we assessed the potential welfare impact of adopting new groundnut spacings among smallholder farmers in northern Ghana. We used data from on-farm experiments, focus group discussions, and a household survey. We applied multiple analytical methods including cost-benefit analysis, adoption prediction model, and economic surplus model. The result of our cost-benefit analysis shows that all spacing options considered in this study are more profitable than the farmers’ practice. Gross financial margin increases from Ghc57/ha in the case of farmers’ practice (i.e., 9 plants/sqm) to Ghc1311/ha in the case of the highest planting density (22 plants/sqm). The benefit-cost-ratio increases from 1.05 under farmers’ practice to 1.87 under the highest plant density option. The maximum adoption rate of the highest and the best planting density (22plants/sqm) is 62% which is expected to be reached within nine years after the initial adoption. Given such a maximum adoption rate and assuming a small open producer economy, the incidence of poverty is expected to decline by about 3.6%. The intervention will also reduce poverty gap and poverty severity which means that poor households will be closer to the poverty line, and their inequality will be reduced. While the impact on welfare remains positive under the closed economy scenario, the magnitudes are not as high as the case of the open economy scenario which implies that smallholder groundnut producers will benefit more if they get access to the international market. The open economy assumption is more plausible than the closed economy one in the context of Ghana since the latter is a temporary barrier that will be lifted by importing countries if conditions are fulfilled. We implicitly assumed in our analysis that existing groundnut growers would continue to grow the crop, but new growers would not come in. However, given the high relative profitability of the new technology, there is a possibility that new growers will enter the market. This implies that the welfare impact of the new technology can be even greater than the figures predicted in this study.

The potential welfare impact of the new technology can be realized: (1) by enhancing the adoption of the technology and (2) by improving farmers’ access to market, particularly the international market. Interventions to improve adoption should aim to achieve two goals i.e., to maximize the number of people adopting the technology (i.e., to achieve high rate of adoption) and to maximize the speed at which the technology will be adopted among most of the population (i.e., to achieve fast diffusion rate). The technology has high relative economic benefit as the result of cost-benefit analysis shows. Moreover, the assessments of the farmers who have directly involved in the trial show that the technology has several other desirable attributes including, among others, low upfront cost associated with it, its high trialability, its observability (the ease with which farmers can gain awareness through local observation), and its reversibility in case farmers would like to restore their traditional practice. These good attributes of the technology together with the relatively high importance of groundnut in the area and other population-level opportunities show that high rate of adoption can be achieved in a short time (possibly within a decade) if the technology is properly promoted. In this regard, integrating the informal information exchange mechanisms (e.g., through farmer-based groups) with formal mechanisms and considering regional differences in extension planning can enhance success. The result of our qualitative assessment also shows that farmers have strong risk minimization motivations and hence promoting risk aversion mechanisms (e.g., weather-index based crop insurance) can enhance adoption. Furthermore, our result shows that probability of adoption declines with the area of land cultivated by farmers. Since farmers cultivating small farmlands are more likely to be poor, addressing adoption constraints specific to them can enhance the overall impact of the technology on poverty. This is particularly important in the situation of closed economy since the rightward shift in market supply of groundnut associated with adoption of the new technology can reduce the income of non-adopter groundnut producers.

Several strategies can be used to improve farmers’ access to (international) market including trade negotiations with importing countries on tariff and non-tariff barriers, taking actions targeted to improve the quality of groundnut grain (such as training farmers on how to prevent aflatoxin and improving storage and transport infrastructure) to meet the phyto-sanitary standards of importing countries. Furthermore, promoting a more integrated groundnut value-chain broadens the demand base (both domestic and international) of the produce. In this regard, policy interventions such as government supports to strengthen processing industries (including community-based processor groups, co-operatives, and individual farmers) and to integrate groundnut producers to the industries (for example through out-grower contracts and membership arrangements) may have a higher and sustainable impact on the welfare of the farmers and beyond. While the open economy policy may improve farmers’ welfare better than the closed one, it should be noted that international prices are volatile and hence a sudden decline in prices in those markets can adversely affect local producers. In fact, the global price for agricultural commodities have shown several significant peaks and troughs in the past decade which could severely affect producers in developing countries [62]. Policy interventions aiming at increasing domestic storage capacity (at producers level and higher levels), value addition, and forward pricing augmented supported by well-functioning market information system can enhance producers’ resilience to shocks arising from price volatilities in international markets.

## Supporting information

S1 File (DOCX)
