# Supplementary material for: Potential impact of groundnut production technology on welfare of smallholder farmers in Ghana
Source: PLoS One. 2022 Jan 14;17(1):e0260877. doi: 10.1371/journal.pone.0260877 (PMC8759684; doi:10.1371/journal.pone.0260877)
Supplement: S1 File — (DOCX) [file pone.0260877.s001.docx]

Table A1. The variables considered in the ADOPT model and mean score

| **Quadrant**  **(A)** | **ADOPT variable**  **(B)** | **Question asked in ADOPT**  **(C)** | **Value range**  **(D)** | **Mean score**  **(E)** |
| --- | --- | --- | --- | --- |
| Relative advantage for the population | 1. Profit orientation | What proportion of the target population has maximizing profit as a strong motivation? | 1(almost none) – 5(almost all) | 4 |
|  | 2. Local community benefit orientation | What proportion of the target households has benefits to their local community/village as a strong motivation? | 1(almost none) – 5(almost all) | 2 |
|  | 3. Risk orientation | What proportion of the target population has risk minimization as a strong motivation? | 1(almost none) – 5(almost all) | 4 |
|  | 4. Enterprise scale | On what proportion of the target farms is there a major enterprise that could benefit from the practice? | 1(almost none) – 5(almost all) | 4 |
|  | 5. Management horizon | What proportion of the target population has a long-term (greater than 10 years) management horizon for their farm? | 1(almost none) – 5(almost all) | 5 |
|  | 6. Short-term constraints | What proportion of the target population is under conditions of severe short-term financial constraints? | 1(almost none) – 5(almost all) | 3 |
| Learnability characteristics of the technology | 7. Trialing ease | How easily can the practice (or significant components of it) be trialed on a limited basis before a decision is made to adopt it on a larger scale? | 1(difficult to try) – 5 (easy to try) | 4 |
|  | 8. Practice complexity | Does the complexity of the practice allow the effects of its use to be easily evaluated when it is used? | 1 (difficult to evaluate)- 5 (easy to evaluate) | 5 |
|  | 9. Observability | To what extent would the practice be observable to farmers who are yet to adopt it when it is used in their district? | 1 (difficult to observe)- 5 (easy to observe) | 5 |
| Population-specific influences on the ability to learn about the technology | 10. Advisory support | What proportion of the target population uses paid advisors capable of providing advice relevant to the practice? | 1(almost none) – 5(almost all) | 4 |
|  | 11. Group involvement | What proportion of the target population participates in farmer-based groups that discuss farming? | 1(almost none) – 5(almost all) | 4 |
|  | 12. Relevant existing skills & knowledge | What proportion of the target population will need to develop substantial new skills and knowledge to use the practice? | 1(almost all) – 5(almost none) | 3 |
|  | 13. Practice awareness | What proportion of the target population would be aware of the use or trialing of the practice in their district? | 1(almost none) – 5(almost all) | 3 |
| Relative advantage of the technology | 14. Relative upfront cost of the practice | What is the size of the up-front cost of the investment relative to the potential annual benefit from using the practice? | 1(very large) – 5(none) | 4 |
|  | 15. Reversibility of the practice | To what extent is the adoption of the practice able to be reversed? | 1(not reversed) – 5(easily reversed) | 4 |
|  | 16. Profit benefit in years that it is used | To what extent is the use of the practice likely to affect the profitability of the farm business in the years that it is used? | -3(large disadvantage) – +4(very large advantage) | 3 |
|  | 17. Profit benefit in future | To what extent is the use of the practice likely to have additional effects on the future profitability of the farm business? | -3(large disadvantage) – +4(very large advantage) | 1 |
|  | 18. Time for profit benefit to be realized | How long after the practice is first adopted would it take for effects on future profitability to be realized? | 1(more than 10 years) – 5(one year) | 5 |
|  | 19. Local village/ community costs and benefits | To what extent would the use of the innovation have net benefits or costs to the local community/village? | -3(large disadvantage) – +4(very large advantage) | 0 |
|  | 20. Time to local village/community benefit | How long after the innovation is first adopted would it take for the expected local village/community benefits or cost to be realized? | 1(more than 10 years) – 5(one year) | - |
|  | 21. Risk | To what extent would the use of the practice affect the net exposure of the farm business to risk? | -3(large increase) – +4(very large decrease) | 1 |
|  | 22. Ease and convenience | To what extent would the use of the practice affect the ease and convenience of the management of the farm in the years that it is used? | 3(large decrease) – +4(very large increase) | 1 |

Table A2: Descriptive statistics of variables included in the logistic regression model

|  | Adopters | Non-adopters | t-value |
| --- | --- | --- | --- |
| Household is headed by male | 0.85 | 0.86 | 0.401 |
| Age of household head (years) | 48.00 | 47.00 | 0.762 |
| Education of household head | 0.26 | 0.22 | 1.101 |
| Household size | 9.92 | 8.54 | 2.328** |
| Area under major cereals (ha) | 3.83 | 2.61 | 2.842*** |
| Area under groundnut (ha) | 1.22 | 0.95 | 2.751*** |
| If groundnut plot is located at homestead | 0.86 | 0.97 | 3.579*** |
| Number of plots | 4.05 | 4.05 | 0.19 |
| Livestock | 4.05 | 2.92 | 1.687* |
| Off-farm activity | 0.74 | 0.66 | 1.906* |
| Credit access | 0.09 | 0.07 | 0.956 |
| Market access | 35.4 | 32.9 | 1.903* |
| Membership in social organization | 0.72 | 0.62 | 2.246** |
| Information access | 0.82 | 0.81 | 0.108 |
| Northern Region | 0.49 | 0.47 | 0.349 |
| Upper West Region | 0.20 | 0.38 | 4.688*** |

Table A3: Changes in income of the sample households at selected predicted adoption levels

|  | Percent of households assumed to be adopting | | |
| --- | --- | --- | --- |
|  | 28% | 55% | 62% |
| Mean per capita income adopter (before adoption) | 1917 | 1847 | 1880 |
| Mean per capita income of non-adopters (before adoption) | 1641 | 1562 | 1453 |
| Open economy case |  |  |  |
| Change in producers surplus for adopters | 76 (4) | 163 (9) | 186 (10) |
| Closed economy case for adopters |  |  |  |
| Change in producers’ surplus | 23 (1.2) | 47 (2.5) | 53 (2.8) |
| Change in consumers’ surplus | 33 (1.7) | 66 (3.6) | 73 (3.9) |
| Total change | 56 (2.9) | 113 (6.1) | 126 (6.7) |
| Non-adopters after adoption |  |  |  |
| Change in producers’ surplus | -38 (2.3) | -71 (4.5) | -61 (4.2) |
| Change in consumers’ surplus | 25 (1.5) | 46 (2.9) | 50 (3.4) |
| Total change | -13 (0.8) | -25 (1.6) | -11 (0.8) |
| Non-producing consumers |  |  |  |
| Total income change | 5 (0.3) | 9 (0.5) | 11 (0.7) |

Note: Figures in parenthesis are percentage values; 28%, 55%, and 62% represent adoption rates at 3years, 6 years, and 9years after the base year, respectively. Non-producing consumers include farmer consumers only since our survey data involved only farmers. In fact, we did not allocate about 20% of the consumers’ surplus. This part of the economic gain is expected to be received by non-farmer consumers.

**Fig A1: Sensitivity analysis regarding peak adoption level of the new groundnut plant spacing**

**Fig A2: Sensitivity analysis regarding time to peak adoption level of the new groundnut plant spacing**
